# Patellar tendon ossification after anterior cruciate ligament reconstruction using bone – patellar tendon – bone autograft

**DOI:** 10.1186/1471-2474-14-164

**Published:** 2013-05-10

**Authors:** Gianluca Camillieri, Vincenzo Di Sanzo, Matteo Ferretti, Cosma Calderaro, Vittorio Calvisi

**Affiliations:** 1 Department of Orthopaedic Surgery, University of L’Aquila, Via Sulbiate 47, Rome, 00188, Italy; 2 Piazzale Salvatore Tommasi 1, Blocco 11, L’Aquila, Fraz. Coppito, 67010, Italy; 3 Department of Surgical Sciences, “Sapienza” University of Rome, Viale Regina Elena 324, Rome, 00185, Italy; 4 Orthopaedic Unit, S. Andrea Hospital, “Sapienza” University of Rome, Via di Grottarossa 1035, Rome, 00189, Italy

**Keywords:** Patellar tendon, Ossification, Anterior cruciate ligament reconstruction, Bone-patellar tendon-bone graft

## Abstract

**Background:**

Among the various complications described in literature, the patellar tendon ossification is an uncommon occurrence in anterior cruciate ligament (ACL) reconstruction using bone – patellar tendon – bone graft (BPTB). The heterotopic ossification is linked to knee traumatism, intramedullary nailing of the tibia and after partial patellectomy, but only two cases of this event linked to ACL surgery have been reported in literature.

**Case presentation:**

We present a case of a 42-year-old Caucasian man affected by symptomatic extended heterotopic ossification of patellar tendon after 20 months from ACL reconstruction using BPTB. The clinical diagnosis was confirmed by Ultrasound, X-Ray and Computed Tomography studies, blood tests were performed to exclude metabolic diseases then the surgical removal of the lesion was performed. After three years from surgery, the patient did not report femoro-patellar pain, there was not range of motion limitation and the clinical-radiological examinations resulted negative.

**Conclusion:**

The surgical removal of the ossifications followed by anti-inflammatory therapy, seems to be useful in order to relieve pain and to prevent relapses. Moreover, a thorough cleaning of the patellar tendon may reveal useful, in order to prevent bone fragments remain inside it and to reduce patellar tendon heterotopic ossification risk.

## Background

Reconstruction of the anterior cruciate ligament (ACL) using bone – patellar tendon – bone graft (BPTB) is a common procedure in orthopaedic surgery but the patellar tendon ossification is an uncommon occurrence. In fact only two cases have been reported in literature [1,2]. We report a case of patellar tendon ossification at 20 months after ACL reconstruction using BPTB.

## Case presentation

**Figure 1:**
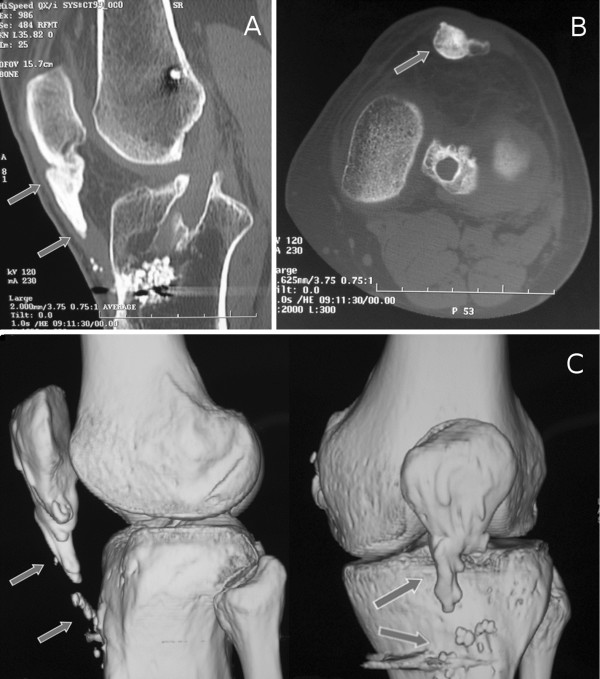
**Computed Tomography (CT) scan image of the patient’s left knee at the time of presentation.** (**A**) Sagittal CT scan image shows a 2.8 × 1.2 cm conical ossification of the proximal patellar tendon (arrows). It is present hyperdense material within the tibial tunnel attributable to a resorbable screw. (**B**) Transverse CT scan image at the joint space level shows patellar tendon ossification (1.5 × 1.2 cm) at the patellar tip (arrows). (**C**) CT 3D reconstruction images show the proximal and the distal patellar tendon ossification morphology (arrows).

**Figure 2:**
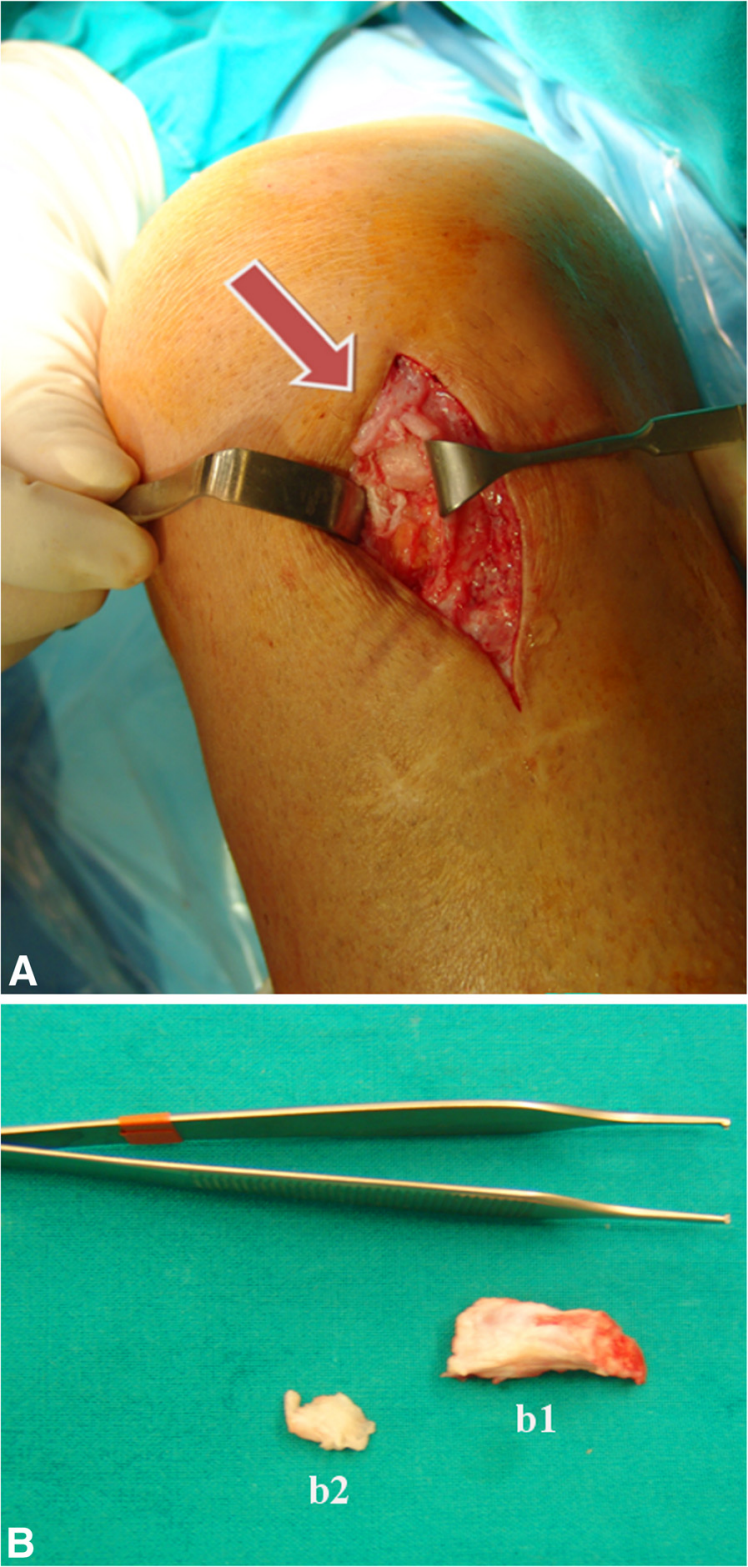
**Intraoperative images.** (**A**) Intraoperative image showing the proximal patellar tendon ossification (arrow). (**B**) Proximal (b1) and distal (b2) removed lesions.

Our patient is a healthy 42-year-old Caucasian man with a history of ACL reconstruction using BPTB performed 20 months before our examination by another surgeon. Ten months from surgery, the patient reported persistent and progressive femoro-patellar pain of the left knee, mainly on the patellar apex, during kneeling, climbing up and down the stairs. He also reported progressive thickening and hard texture of the patellar tendon compared to the contralateral. He did not report other knee injuries after the ACL reconstruction. At clinical examination, we noticed two prominences with hard texture, more evident in 90° knee flection, in lateral profile. One prominence was localized on the proximal portion of the patellar tendon and another smaller on the distal portion. The range of motion was limited by pain at 0°-110°, Lachman test, anterior drawer, Jerk test, Pivot-shift and meniscal tear tests were negative, IKDC score (International Knee Documentation Committee) was 41.1. The ultrasound examination was performed at first and the images showed two bone-like echogenic lesions with acoustic shadowing behind. The radiological images revealed two ossifications localized on proximal and distal portion of the patellar tendon. The Computed Tomography (CT) study (Figure 1A, B) was performed and it showed a 2.8 × 1.5 × 1.2 cm conical ossification of the proximal portion and a 1 × 1 × 0.7 cm ossification of the distal portion of the patellar tendon, well represented in the 3D reconstructions (Figure 1C). A comprehensive metabolic panel (glucose, lipids, albumin, total proteins, creatinine, blood urea nitrogen, alanine amino transferase, aspartate amino transferase, alkaline phosphatase, bilirubin, sodium, potassium, chloride and calcium) was performed to exclude metabolic diseases and the result of all tests was within normal ranges. Our hypothesis was that the ossification was the consequence of the persistence of bone fragments inside the patellar tendon after the closure of the soft tissues. Unfortunately, the patient had not any post-operative radiographs to prove this hypothesis. Two months after the diagnosis of ossification of the patellar tendon, an open surgical treatment was performed to remove the proximal and distal lesions (Figure 2A, B). Two masses of bone texture, localized at the patellar apex and at the distal portion of the tendon, inside the tendon sheat and surrounded by tendon’s fibers, were removed. A rigid orthesis locked in extension was applied and an anti-inflammarory therapy with ibuprofen (200 mg twice a day for a week) was performed. The rehabilitation program consisted in: during the I-II weeks loading according the pain, isometric contraction of the quadriceps three times a day, flection 0°-90° twice a day using a CPM device (Continuous Passive Motion) and flexors stretching; during III-IV weeks were added isotonic contraction of the quadriceps 0°-30° and active/passive flection 0°-120° (III-IV week) and 0°-135° (V-VI week). After six weeks of rehabilitation the patient returned to normal activity. At three years from surgery, the patient did not report femoro-patellar pain, there was not range of motion limitation, IKDC score was 90.8. Both clinical and radiological examinations resulted negative too.

## Conclusions

There is no unanimous opinion in literature on the causes and the treatment of the patellar tendon ossification but a traumatic etiology seems to be the most affordable hypothesis. In literature the ossification of the patellar tendon has been reported mainly related to knee injuries [3,4] or as a consequence of total/partial patellectomy [5,6] and intramedullary nailing of the tibia [7-9]. A rare and interesting report was described by Chen et al., an extensive heterotopic ossification after patellar tendon repair in a 32-year-old African-American man with a rare trisomy 8 mosaicism. These authors suggested an association between trisomy 8 mosaicism and increased risk of heterotopic ossification [10].

As our case, two authors previously reported symptomatic patellar tendon ossification following the reconstruction of the ACL using BPTB [1,2]. Valencia and Gavìn first reported a case of ossification of the proximal 2 cm of the patellar tendon after ACL reconstruction with BPTB. The patient was asymptomatic until the eighth postoperative month, when he started complaining of pain in the proximal insertion area of the patellar tendon. The clinical and radiological examinations showed ossification of the proximal third of the patellar tendon that was removed by surgery. A postoperative rehabilitation based on early mobilization and a 6 week anti-inflammatory therapy with indomethacin were done [1]. Six months later the patient was free of pain without range of motion limitation.

Recently, Erdil et al. described another case of symptomatic ossification of the patellar tendon after ACL surgery with BPTB in a 36-year-old man. The patient had range of motion limitation and pain occurred six weeks after the ACL reconstruction. The clinical and radiological examinations showed the ossification of the proximal half of the patellar tendon. The first strategy was based on a conservative treatment using physical therapy and a rehabilitation program consisting of mobilization and achievement of normal range of motion. One year later, as the persistence of the ossification and the pain, the lesion was surgical removed. Surgery was followed by indomethacin therapy for 6 weeks and rehabilitation to recover active/passive range of motion (0°-135°) and muscle strength. The examination at 36 month follow-up revealed the achievement of full range of motion without recurrences [2].

A similar case was described by Erdogan et al. who reported a case of patellar tendon calcification (not ossification) after ACL reconstruction with BPTB [11]. According to Erdogan, the calcification was due to the retention of bone debris within the patellar tendon during the graft harvest and tunnel reaming. Moreover these authors believed that an aggressive rehabilitation program in the immediate postoperative period may subject the tendon to excessive loads resulting in microtrauma with focal degeneration followed by calcification [11].

In our opinion to reduce patellar tendon heterotopic ossification risk in ACL surgery with BPTB, a thorough cleaning of the patellar tendon may reveal useful in order to prevent bone fragments persistence inside it. Moreover, we suggest that, at the patellar and tibial bone defect sites, the soft tissues and the periostium should be used to prepare a containment floor to avoid dispersion of bone fragments.

According to the literature, the surgical removal of ossifications followed by anti-inflammatory therapy and an immediate rehabilitation protocol for range of motion recovery, seems to be useful in order to relieve pain and to prevent relapses of ossification.

### Patient consent

“Written informed consent was obtained from the patient for publication of this Case report and any accompanying images. A copy of the written consent is available for review by the Editor of this journal.”

## Abbreviations

ACL: Anterior cruciate ligament; BPTB: Bone-patellar tendon-bone; CT: Computed tomography; CPM device: Continuous passive motion; IKDC: International knee documentation committee.

## Competing interests

The authors declare that they have no conflict of interest and sources of financial support to the publication of this article.

## Authors’ contributions

GC: Conception and design, data collection, critical revision of the manuscript, supervision. VDS: Analysis and interpretation, writing the manuscript. MF: Analysis and interpretation, writing the manuscript. CC: Analysis and interpretation, writing the manuscript. VC: Conception and design, critical revision of the manuscript, supervision. All authors read and approved the final manuscript.

## Pre-publication history

The pre-publication history for this paper can be accessed here:

http://www.biomedcentral.com/1471-2474/14/164/prepub
